# Chagas disease and amiodarone: a bibliometric and systematic review from cell to patient

**DOI:** 10.3389/fphar.2026.1749345

**Published:** 2026-04-02

**Authors:** Juliana Magalhães Chaves Barbosa, Raquel Barreto Duarte, Henrique Horta Veloso, Anissa Daliry, Kelly Salomão

**Affiliations:** 1 Cell Biology Laboratory, Oswaldo Cruz Institute, Oswaldo Cruz Foundation, Rio de Janeiro, Brazil; 2 Laboratory of Clinical and Experimental Physiopathology, Oswaldo Cruz Foundation, Rio de Janeiro, Brazil; 3 Evandro Chagas National Institute of Infectious Diseases, Oswaldo Cruz Foundation, Rio de Janeiro, Brazil

**Keywords:** amiodarone, bibliometric and systematic review, cardiomyopathy, Chagas disease, *Trypanosoma cruzi*

## Abstract

**Background:**

Chagas disease (CD) is a neglected tropical illness caused by *Trypanosoma cruzi*, with a high prevalence in Latin America. Arrhythmias are common in patients with Chagas cardiomyopathy, and amiodarone (AMIO) has been widely used in their management. Recent studies have also suggested a potential role for AMIO as a trypanocidal agent. This review aims to evaluate the current evidence regarding AMIO therapy for the treatment of patients with CD.

**Methodology:**

This study combines bibliometric and systematic review approaches to explore the use of AMIO in the treatment of CD. The literature search was conducted in PubMed. Bibliometric analyses were performed using the Bibliometrix 2.2.1 package in R 3.6 (R Core Team, 2019). Relationship mapping was carried out using VOSviewer 1.6.16 (https://www.vosviewer.com/) to visualize bibliographic network structures. The systematic review component followed the Preferred Reporting Items for Systematic Reviews (PRISMA) guidelines.

**Principal Findings:**

A total of 52 original articles published in 35 journals were included, involving contributions from 269 authors, predominantly from Latin America. Brazil was the leading contributor, followed by Venezuela, the United States, Argentina, and Spain. The bibliometric analysis identified several emerging trends: (1) treatment outcomes such as mortality and hospitalization; (2) the antiarrhythmic effects of AMIO; (3) potential trypanocidal effects of AMIO; (4) the use of AMIO in combination with other drugs for etiological treatment; and (5) its possible anti-inflammatory effects.

**Conclusion:**

This review highlights a significant gap in literature, specifically the lack of rigorous clinical studies evaluating the impact of AMIO in patients with chronic Chagas cardiomyopathy (CCC). Additionally, we identify promising avenues for future research to better understand AMIO’s therapeutic role in the management of CD.

## Introduction

Chagas disease (CD), also known as American trypanosomiasis (Lidani et al., 2019), remains a major public health challenge, particularly in Latin America, where it continues to contribute significantly to morbidity and mortality (Marin-Neto et al., 2023). CD affects an estimated 7 million people worldwide (World Health Organization, 2025), including populations in non-endemic regions such as Europe, North America, Japan, and Australia. This global spread is largely attributed to increased migration from endemic areas to high-income countries in recent decades (Lidani et al., 2019).

Clinically, CD is divided into two distinct phases: acute and chronic. During the acute phase, most individuals experience mild, self-limiting symptoms that often go undetected in clinical settings. This phase typically lasts four to 8 weeks, after which the infection progresses to the chronic stage (Marin-Neto et al., 2023; Bern, 2015). In the absence of effective treatment during the acute phase, the infection can persist for life. Alarmingly, fewer than 1% of affected individuals have access to proper diagnosis and treatment (Chatelain, 2016). Chronic *T. cruzi* infection without clinical manifestations of CD is classified as the indeterminate form. It is estimated that 20%–30% of individuals with this form will, over several decades, develop clinically manifest cardiac disease, gastrointestinal complications, or both (Marin-Neto et al., 2023; Bern, 2011).

Chronic Chagas cardiomyopathy (CCC) is characterized by myocardial inflammation and fibrosis, which contribute to increased myocardial stiffness and both right and left ventricular dysfunction. This pathophysiological process ultimately leads to severe dilated cardiomyopathy accompanied by arrhythmias (Marin-Neto et al., 2023; Nunes et al., 2018). Since trypanocidal treatment with benznidazole (Bz) has not demonstrated efficacy in slowing cardiac deterioration in patients with CCC during clinical trials (Morillo et al., 2015), current management focuses on controlling heart failure and arrhythmia to slow disease progression (Marin-Neto et al., 2023). According to the Brazilian Society of Cardiology (SBC) Guideline on the Diagnosis and Treatment of Patients with Cardiomyopathy of Chagas Disease, amiodarone (AMIO) is the most recommended antiarrhythmic agent for the pharmacological management of cardiac arrhythmias in CCC (Marin-Neto et al., 2023).

AMIO is a benzofuran compound derived from the plant *Ammi visnaga*, a medicinal herb traditionally used in various ancient civilizations, including by the Persian physician Avicenna in the 10th century AD, for the treatment of conditions such as asthma, chest pain, and menstrual disorders (Tavolinejad et al., 2019). AMIO was first synthesized in 1961 for use as an antianginal agent. Its antiarrhythmic activity was discovered in animal models in 1969 (Charlier et al., 1969), and its clinical use as an antiarrhythmic drug was first reported in 1974. Throughout the 1970s, both intravenous and oral formulations were tested in clinical settings, leading to its approval by the US Food and Drug Administration in 1985 (Tavolinejad et al., 2019). Mechanistically, AMIO acts primarily by modulating cardiac ion channels - including sodium, potassium, and calcium channels -prolonging action potential duration and refractory periods, which underlies its antiarrhythmic effects (Charlier et al., 1969). The use of AMIO in patients with CCC has been documented since the early 1980s (Greco et al., 1980; Rosenbaum et al., 1983). However, clinical studies specifically focused on this population have been limited, and their findings remain insufficient to guide evidence-based practice (Marin-Neto et al., 2023; Stein et al., 2018).

**FIGURE 1:**
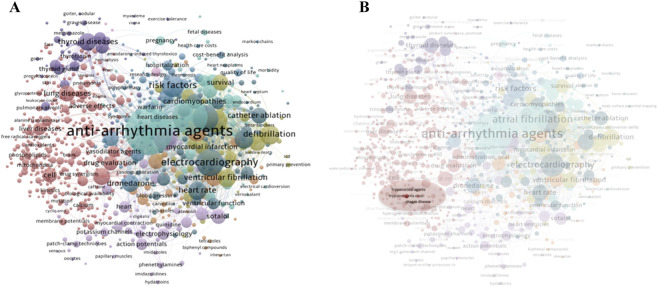
Bibliographic network of publications related to “Amiodarone” from 1990–2024 (9,983 articles), based on keyword co-occurrence analysis in MEDLINE (accessed via PubMed). The visualization was generated using VOSviewer 1.6.16. Keywords with a minimum occurrence of ten were included in the analysis. **(A)** Terms directly related to “Amiodarone”, as well as those referring to countries, methodologies, study types, ethnicity, and age groups, were excluded to improve clarity; **(B)** On the right side of the network, key terms of interest - including “trypanocidal activity”, “Chagas disease” and “*Trypanosoma cruzi*” are highlighted in red.

In 2006, Benain and colleagues reported for the first time the anti-*T. cruzi* activity of AMIO, increasing interest in its potential role in treating CCC. Its trypanocidal effect is believed to result from the disruption of Ca^2+^ homeostasis and inhibition of oxidosqualene cyclase in *T. cruzi* (Benaim et al., 2006). As a result, research exploring the use of AMIO as an etiological treatment of CD has gained prominence in the scientific literature, as illustrated in Figure 1.

Our aim was to conduct a combined bibliometric and systematic review to evaluate the effects of AMIO on cardiac arrhythmias, mortality, parasitic load, and immune response in patients with CD. Additionally, we sought to explore the potential benefits and risks associated with AMIO use, assess its possible role in combination therapies, and identify gaps in the current evidence that warrant further investigation.

## Methods

### Type of study

This work is a combined bibliometric and systematic review of the relationship between AMIO and CD. Data on authorship, countries of origin, affiliated institutions, and health-related descriptors were analyzed, along with scientific output over time.

### Protocol

The review was conducted in accordance with the Preferred Reporting Items for Systematic Reviews (PRISMA) guidelines (Page et al., 2021) and followed the methodological recommendations of the Cochrane Handbook for Systematic Reviews of Interventions (Cumpston et al., 2019) (see Supplementary Table S1). Although the review protocol was not preregistered, the methodological procedures were defined *a priori* and consistently applied throughout the study.

### Search strategy and database survey

**FIGURE 2:**
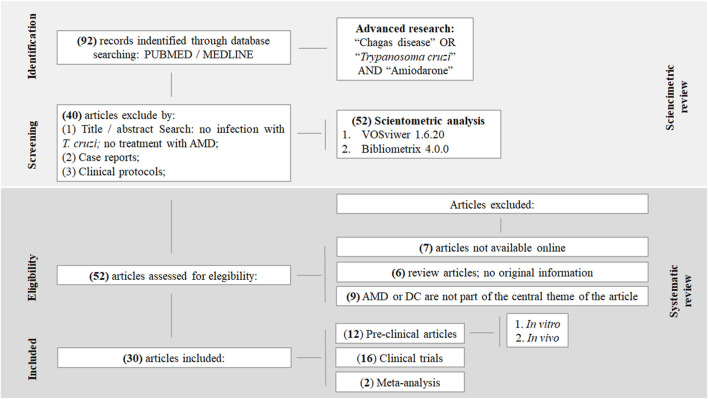
Flow diagram illustrating the selection process for the bibliometric and systematic review.

The search was conducted using the MEDLINE database accessed via PubMed (https://pubmed.ncbi.nlm.nih.gov/). The search period was defined from 1980, corresponding to the year of publication of the earliest article identified on this topic using the predefined search strategy, through 15 December 2024, the date of the last database search. The search strategy used text words (“Chagas disease” OR “*Trypanosoma cruzi*”) AND (“Amiodarone”) to ensure comprehensive coverage of all relevant studies, including the most recent publications that had not yet been indexed with MeSH terms. No language restrictions were applied. (Figure 2). The population of primary interest was human patients with CD, while preclinical and *in vitro* studies were also included to provide mechanistic insights and support the translational understanding of AMIO.

### Eligibility criteria and study selection

Titles and abstracts retrieved from the database were independently reviewed by two researchers (J.M.C.B. and K.S.). Abstracts lacking sufficient information to apply the eligibility criteria were selected for full-text evaluation.

In the first phase, articles were excluded if they met any of the following criteria: (1) title or abstract was unrelated to *T. cruzi* infection or AMIO treatment; (2) the article was a case report; or (3) the article was a clinical report. Articles selected during this phase were included in the bibliometric analysis.

In the second phase, the same reviewers independently evaluated the full texts of the remaining articles. The following exclusion criteria were applied: (1) full text was not available online, as access to complete content was required for the bibliometric analysis; (2) review articles containing no original data; or (3) articles in which AMIO or CD were not the central focus (Figure 2). Articles meeting the inclusion criteria in this phase were included in the systematic review. Any discrepancies between reviewers were resolved by consensus or adjudicated by a third reviewer (A.D.). Additionally, references cited within the selected articles were screened to identify relevant studies not captured in the initial search.

### Bibliometric analysis

Bibliometric data were analyzed using the Bibliometrix 2.2.1 package (Aria and Cuccurullo, 2017) implemented in R version 3.6 (R Core Team 2019). Relationship mapping and network analysis were performed using VOSviewer version 1.6.16 (https://www.vosviewer.com/), which constructs bibliographic networks based on co-authorship, keyword co-occurrence, and citation data (Van Eck and Waltman, 2010). The “thesaurus” tool in VOSviewer was used to consolidate synonymous terms.

We examined temporal trends in publication volume and identified the most prolific countries, journals, and authors. International collaboration networks were visualized to highlight inter-country relationships. The Dominance Factor of authors was calculated using the formula: DF = number of first-authored articles/(total number of authored articles - number of single-authored articles). A higher DF indicates a greater prevalence of first authorship among multi-authored papers, reflecting the author’s leadership role in collaborative publications (Kumar et al., 2019).

## Results

### Bibliometric review

A total of 92 articles were initially retrieved from the MEDLINE database (accessed via PubMed). After applying the predefined exclusion criteria (Figure 2), 52 articles were selected for inclusion in the bibliometric analysis (Supplementary Table S1). The earliest publication was from 1980, so the time frame analyzed spanned from 1980 to December 2024. During this period, the field exhibited an average annual growth rate of 1.59%, with a mean publication output of 1.18 articles per year (Table 1).

**TABLE 1:** Main information about data of topic search: (Chagas disease OR *Trypanosoma cruzi*) AND (Amiodarone), in PUBMED.

Main information about data	
Timespan	1980:2024
Sources (journals, books, etc.)	35
Documents	52
Annual growth rate %	1.59
Document average age	18
Document contents	
Keywords plus (ID)	295
Authors	269
Authors collaboration	
Single-authored docs	0
Co-authors per doc	6.6
International co-authorships %	15.38

Keywords Plus (ID): Terms automatically generated from cited references’ titles, extending author keywords; IDs, are unique identifiers for bibliometric analysis in *bibliometrix*.

**FIGURE 3:**
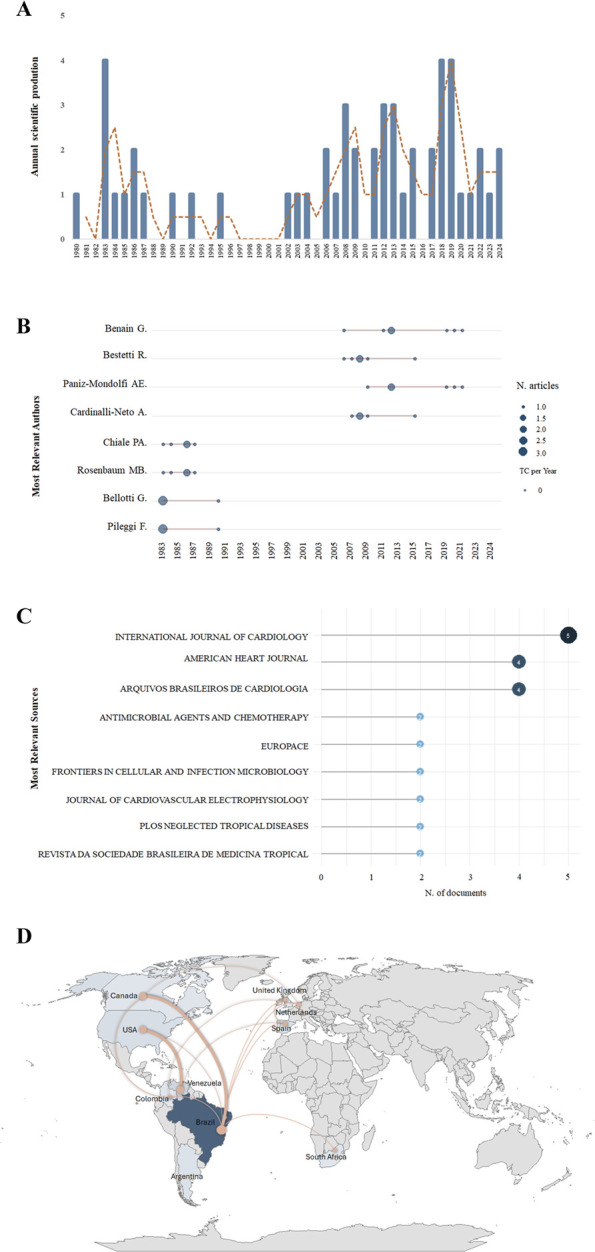
Trends in publications related to CD and AMIO from 1980 to 2024. **(A)** Annual distribution of publications with a trend line (dotted orange line); **(B)** Frequency distribution of publications by the nine most productive authors in the field; **(C)** Frequency distribution of publications by the nine most productive scientific journals; and **(D)** Institutional and geographical patterns of authorship. The blue gradient indicates the scientific productivity of each country (darker shades represent higher productivity). Orange lines represent international collaborations, with line thickness proportional to the frequency of collaborative publications.

Although the number of publications increased over time, the growth showed modest fluctuations (Figure 3A). Two distinct periods of increased scientific production were identified: 1983–1986 and 2006 to the present (Figure 3A).

A total of 269 authors contributed to the selected publications, with no single-authored documents. The top five most prolific contributors to this research were: (1) Benain G. from the *Instituto de Estudios Avanzados, Venezuela* (7 articles; DF: 0.85); (2) Bestetti R. B. from *Hospital de Base da Faculdade de Medicina de São José do Rio Preto*, Brazil (6 articles; DF: 0.50); (3) Paniz M., also from the *Instituto de Estudios Avanzados*, Venezuela (6 articles; DF: 0.16); (4) Cardinalli-Neto A. from *Hospital de Base de São José do Rio Preto*, Brazil (5 articles; DF: 0.40); (5) Rosenbaum M. B. from *Hospital Ramos*, Argentina (5 articles; DF: 0.40); and (6) Chiale P. A. from *Hospital Ramos Mejia*, Argentina (5 articles; DF: 0.20) (Figure 3B). Notably, all these highly productive authors are based in countries endemic for CD, highlighting that research activity on this topic is concentrated in regions with direct epidemiological relevance (World Health Organization, 2025). This geographic concentration likely reflects local research priorities, access to patient populations, and potential variations in parasite strains, which may influence study design and findings (Marin-Neto et al., 2023).

The journals publishing the greatest number of articles on this topic are shown in Figure 3C, and their respective impact factors are presented in Tables 1, 2. First-author affiliation was also analyzed, revealing that most publications originated from Brazilian institutions, followed by those in Venezuela and the United States (Figure 3D). The *Instituto Oswaldo Cruz* (IOC), Brazil, was the most productive institution (contributing 11% of all studies), followed by the *Universidade Federal de Minas Gerais* (UFMG) and *Instituto D’OR de Pesquisa e Ensino* (IDOR), also in Brazil. As a result, Brazil emerged as the leading country in terms of publication volume, followed by Venezuela, the USA, Argentina, and Spain (Figure 3D). In terms of international collaboration, the most frequent partnerships occurred between Brazil–Canada, Brazil–USA, Venezuela–Poland, and Venezuela–USA (Figure 3D).

**TABLE 2:** Preclinical studies evaluating amiodarone in Chagas disease.

Author,Year, doi	IF*	Type of study	Experimental model	*T. cruzi* strain	Intervention	Phase of DC	Combined tratment	Findings
Benaim et al. (2006) doi: 10.1021/jm050691f.	Journal of Medicinal Chemistry (7,3)	*In vitro / In vivo*	Infected Vero cells / infected female NMRI-IVIC mice	The EP stock of *T. cruzi* (a virulent strain isolated)	Oral treatment at every other day for amiodarone (50 mg/kg), during 30 days.	Acute	Posaconazole (20 mg/kg/day)	(1) AMD disrupts Ca2+ homeostasis and blocks sterol biosynthesis in *T. cruzi*; (2) AMD has direct in vivo activity in acute murine models of CD and (3) There was a synergistic effects of AMD and Posaconazole against parasite.
Adesse et al. (2011) doi: 10.1128/AAC.01129-10.	Antimicrobial Agents and Chemotherapy (4,1)	*In vitro*	Infected cardiac cells	Y	Treatment with AMD (2.5 to 10 µM) in infected-*T. cruzi* cardiac cells.	-	-	(1) AMD has a selective antiproliferative effect on *T. cruzi* in infected-cardiac cells; (2) AMD induced ultrastructural damage to intracellular amastigotes (mitochondrial swelling, reservosomes and kinetoplast disorganisation) but promoted a structural and functional recovery of the host cells.
Benaim et al. (2012) doi: 10.1128/AAC.00207-12	Antimicrobial Agents and Chemotherapy (4,1)	*In vitro*	Infected Vero cells	CL Brener	Treatment with AMD (at different concentrations) in infected-*T. cruzi* Vero cells and epimastigotes.	-	-	In comparasion with AMD, dronedarone (an amiodarone derivative) was higher trypanocidal effect.
Veiga-Santos et al. (2012) doi: 10.1016/j.ijantimicag.2012.03.009.	International Journal of Antimicrobial Agents (4,9)	*In vitro*	Infected peritoneal macrophages	Y	Treatment with AMD (4 to 20 µM) in infected-*T. cruzi* peritoneal macrophages	-	Posaconazole (1 to 20 nM)	(1) Intracellular amastigotes treated with AMD exhibited autophagosomes, projections of the plasma membrane, disruption of the Golgi complex and accumulation of lipid bodies; (2) Confirmed the synergism action mechanisms of Posaconazol and AMD against *T. cruzi* and reported that combination of drugs were related with autophagic death of the intracellular amastigote.
Bellera et al. (2013) doi: 10.1021/ci400284v.	Journal of Chemical Information and Modeling (5,6)	*In vitro*	Epimatigotes	Y	*T. cruzi* epimastigotes were grown in the absence or presence of AMD (0−200 µM).	-	-	The antitrypanosomal effect of AMD, at least partially, is related to inhibition of Cruzipain activity.
Lourenço et al. (2018) doi: 10.1590/0037-8682-0285-2017.	Revista da Sociedade Brasileira de Medicina Tropical (1,1)	*In vitro*	Epimatigotes	Y	*T. cruzi* epimastigotes were grown in the absence or presence of AMD (2 to 20 mg ml-1).	-	Benznidazole (2 to 20 mg ml-1)	(1) AMD had equivalent trypanocidal activity to Benznidazole at all doses studied; (2) There was no pharmacological interaction between AMD and Bz.
Madigan et al. (2019) doi: 10.2460/javma.255.3.317.	Journal of The American Veterinary Medical Association (1,6)	*In vivo*	Naturally infected dogs	-	2 protocols: (1) Oral treatment of AMD 30 mg/kg/day for 7 days, plus 15 mg/kg/day for 14 days, plus 7.5 mg/kg/day for 12 months; (2) oral treatment of AMD 7.5 mg/kg/day for 12 months.	Chronic	Itraconazole (10 mg/kg/day) for 12 months.	(1) Treatment with AMD plus Itraconazole improved some of the echocardiographic (increased fractional shortening, elimination of valvular regurgitation, reduction of ventricular wall thickening, and enhanced septal wall kinesis), which suggested improvement in myocardial function in infected-dogs; (2) Combined treatment enhence the survival rates.
Zao et al. (2019) doi: 10.1177/1040638719868508	Journal of Veterinary Diagnostic Investigation (1,5)	*In vivo*	Naturally infected dogs	-	Oral treatment of AMD 7.5 mg/kg /day for 1 year	Chronic	Itraconazole (10 mg/kg/day) for 1 year	Treatment with AMD plus Itraconazole generates low or undetectable levels of parasitemia mensure by multiple blood collections and PCR re-testing, using nDNA- and kDNA-based rtPCR methods in parallel.
Sass et al. (2019) doi: 10.4269/ajtmh.19-0023.	American Journal of Tropical Medicine and Hygiene (1,8)	*In vitro*	Infected cardiomyocytes (hiPSC-CMs)	I or II strains	Treatment with AMD and Itraconazole (at different concentrations) in infected-*T.cruzi* hiPSC-CMs.	-	Itraconazole	Combination of Itraconazole and AMD were more effective against *T. cruzi* than the single substances, or Bz.
Barbosa et al. (2022a) doi: 10.1128/spectrum.01852-21.	Microbiology Spectrum (3,7)	*In vivo*	Infected male Swiss mice	Y	Oral treatment of AMD 50 mg/kg/day for 5 days	Acute	Benznidazole (25 mg/kg/day) for 5 days	Combination of Bz and AMD improved: (1) parasitism elimination, (2) mouse survival, (3) conexin-43 expression in heart tissue; and reduce: (4) heart inflammation, such as MCP-1, Il-6 and TNF levels and (5) cardiac electrical abnormalities.
Barbosa et al. (2022b) doi: 10.3389/fcimb.2022.975931.	Frontiers in Cellular and Infection Microbiology (4,6)	*In vitro*	Infected cardiac cells	Y	Treatment with AMD and Bz (at different concentrations) in infected-*T. cruzi* cardiac cells and trypamastigotes.	-	Benznidazole (5 to 20 µM)	(1) Combination of Bz and AMD did not interfere with the trypanocidal efficacy of each drug alone against the relevant parasite forms for mammalian host infection; (2) The combined treatment of *T. cruzi*-infected cardiac cells seems to exert a cardioprotective being more effective in recovering the damage to the host cell cytoskeleton.
Barbosa et al. (2024) doi: 10.1016/j.biopha.2024.116742.	Biomedicine & Pharmacotherapy (7,2)	*In vivo*	Infected female C57BL/6 mice	Colombian	Oral treatment of AMD 50 mg/kg/day for 30 days	Chronic	Benznidazole (25 mg/kg/day) for 30 days	Combination of Bz and AMD proved to be the most effective in mitigating the ventricular dysfunction caused by the infection, while also modulating crucial pathogenic factors of CCC, including TNF production, ROS levels, fibronectin deposition and, Cx-43 expression in cardiac tissue.

*Impact factors are based on data from 2024.

**FIGURE 4:**
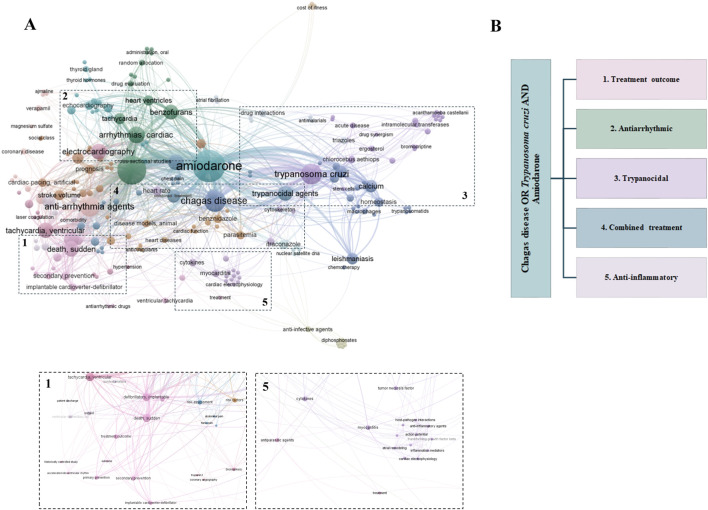
Keyword relationships and thematic mapping were based on publications retrieved from MEDLINE (PubMed) using the topic search (Chagas disease OR *Trypanosoma cruzi*) AND (Amiodarone), 1980–2024 (92 articles). A minimum threshold of one occurrence per keyword was applied. Keywords related to countries, methodologies, study types, ethnicity, and age were excluded. **(A)** Network visualization of keyword co-occurrence generated using VOSviewer, with a segmented keyword network corresponding to the thematic areas and **(B)** identification of five thematic clusters that guided the systematic review.

To better understand research trends and thematic focus areas, a keyword analysis was performed. Synonyms and singular/plural variations were manually consolidated, resulting in a total of 224 keywords. VOSviewer was then used to calculate keyword frequency and visualize co-occurrence patterns (Figure 4A). Based on this analysis, five major research themes emerged, which guided the structure of the systematic review (Figure 4B): (1) treatment outcomes (e.g., mortality and hospitalization, broadly defined as any unplanned admission to a healthcare facility due to clinical complications), (2) antiarrhythmic effects of AMIO, (3) trypanocidal effect of AMIO, (4) combination therapy involving AMIO and other trypanocidal drugs, and (5) potential AMIO anti-inflammatory effects. These five thematic areas are subsequently examined in depth in the following section, corresponding to the systematic review (Figure 4B).

## Systematic review

### Treatment outcomes: mortality and hospitalization

We included seven articles that evaluated the impact of AMIO treatment on critical endpoints - namely, mortality and hospitalization - in patients with CCC (Stein et al., 2018; Ayub-Ferreira et al., 2013; Fortes Silva et al., 2018; Carmo et al., 2018; Gali et al., 2014; Martinelli-Filho et al., 2024; Leite et al., 2003). Although robust meta-analyses have demonstrated that AMIO significantly reduces total mortality and sudden cardiac death in cardiopathies of other etiologies (e.g., the ATMA study; Sim et al., 1997), its survival benefit in CCC remains inconclusive (Effect of prophylactic amiodarone on, 1997; Sim et al., 1997).

(1) Ayub-Ferreira et al. (2013) found that AMIO use was an independent risk factor for mortality in patients with CCC. However, the study emphasized that AMIO usage in the cohort did not align with guideline-based heart failure management, warranting careful reevaluation of these findings (Ayub-Ferreira et al., 2013). In contrast, **(2)** Fortes Silva et al. (2018) observed that AMIO therapy improved heart rate variability (HRV), indicating a shift in sympatho-vagal balance toward parasympathetic predominance. This autonomic modulation suggests a potential cardioprotective effect that could translate into improved patient survival (Fortes Silva et al., 2018).

(3) Carmo et al. (2018), through a pooled data analysis, reported no significant difference in mortality between patients treated with implantable cardioverter-defibrillators (ICDs) and those treated with AMIO alone (Carmo et al., 2018). Interestingly, (4) Gali et al. (2014) demonstrated that the combination of ICD and AMIO therapy significantly reduced all-cause mortality and sudden cardiac death compared to AMIO monotherapy in CCC patients with life-threatening ventricular arrhythmias (Gali et al., 2014). Recently, (5) Martinelli-Filho et al. (2024) found that although ICD therapy did not reduce all-cause mortality compared to AMIO, it significantly lowered the incidence of sudden cardiac death, pacing requirements, and heart failure-related hospitalizations (Martinelli-Filho et al., 2024).

Furthermore, the use of different antiarrhythmic agents was also compared for hard endpoints. **(6)** Leite et al. (2003) compared AMIO with sotalol in patients with CCC and reported no statistical difference in total mortality between the two treatment groups (40.2% with AMIO vs. 36.0% with sotalol) (Leite et al., 2003). Finally, **(7)** Stein et al. (2018) conducted a meta-analysis and concluded that, despite AMIO’s efficacy in reducing ventricular arrhythmias, there is no clear evidence that it reduces sudden death or hospitalization in patients with CCC (Stein et al., 2018).

Overall, the evidence indicates that AMIO may reduce the incidence of sudden cardiac death and heart failure-related hospitalizations in patients with CCC, particularly when combined with device therapy; however, the survival benefit remains inconclusive, and the quality of evidence is limited, highlighting the need for further robust clinical studies.

### Arrhythmias

Cardiac arrhythmias are a hallmark of CCC (Marin-Neto et al., 2023). Electrocardiographic (ECG) abnormalities are highly prevalent in individuals seropositive for *T. cruzi*. The most commonly observed alterations include atrioventricular (AV) conduction blocks, right bundle branch block, left anterosuperior fascicular block, ventricular repolarization disturbances, and ventricular ectopic beats (Marin-Neto et al., 2023; Grupi et al., 1995). Of particular concern are ventricular arrhythmias, such as polymorphic ventricular extrasystoles (VEs) and ventricular tachycardia (VT), which predict syncope and sudden cardiac death due to ventricular fibrillation (VF) (Ribeiro et al., 2014). Importantly, malignant ventricular arrhythmias are significantly more frequent in CCC than in other cardiomyopathies (Cardinalli-Neto et al., 2006; Martinelli Filho et al., 2000).

Three preclinical studies assessed the antiarrhythmic potential of AMIO in animal models of CD (Madigan et al., 2019; Barbosa et al., 2022a; Barbosa et al., 2024) (Table 2). (1) Madigan et al. (2019) investigated the combination of AMIO and itraconazole in naturally *T. cruzi*-infected dogs. The combined treatment significantly normalized ECG parameters; however, the study did not isolate the effect of AMIO, limiting conclusions about its standalone antiarrhythmic efficacy (Madigan et al., 2019). (2) Our group, in Barbosa et al. (2022a), using a mouse model of acute *T. cruzi* infection, showed that AMIO prevented increases in P wave and QRS interval duration, which are markers of conduction abnormalities. Nevertheless, AMIO treatment was associated with a higher incidence of bradycardia (Barbosa et al., 2022a). (3) In Barbosa et al. (2024), we also evaluated AMIO in a mouse model of chronic *T. cruzi* infection. Paired analysis (pretreatment versus post-treatment) revealed that AMIO reversed prolonged QTc intervals and reduced the incidence of first- and second-degree AV conduction blocks. Again, a significant increase in bradycardia was noted with AMIO use (Barbosa et al., 2024). Overall, the consistency between findings from preclinical and clinical studies reinforces the translational value of these animal models in addressing knowledge gaps related to AMIO’s role in CCC management.

We identified eight clinical studies assessing AMIO’s effects on ventricular arrhythmias in CCC patients (Rosenbaum et al., 1983; Stein et al., 2018; Bellotti et al., 1983; Chiale et al., 1984; Carrasco et al., 1985; Haedo et al., 1986; Rosenbaum et al., 1987; Cardinalli-Neto et al., 2015) (Table 3). (1) Bellotti et al. (1983) reported suppression of ventricular extrasystoles (VEs) in 83.3% of patients and VT in 33.3% following AMIO therapy (Bellotti et al., 1983). In the same year, (2) Rosenbaum et al. (1983) reported complete suppression of both VEs and VT with AMIO (Rosenbaum et al., 1983). In 1984, (3) Chiale et al. (1984) observed an antiarrhythmic response in 91% of patients, with a notable reduction in recurrences of VF and VT episodes (Chiale et al., 1984). In 1985 and 1986, (4) Carrasco et al. (1985) and (5) Haedo et al. (1986) reiterated the efficient antiarrhythmic effect of AMIO in CCC patients. Carrasco et al. demonstrated complete suppression of ventricular arrhythmias in 67% of patients treated with AMIO (Carrasco et al., 1985). Haedo et al. compared the efficacy of four antiarrhythmic drugs: verapamil, 17-monochlor-acetylajmaline, mexiletine, and AMIO, concluding that AMIO was the most effective agent, especially against life-threatening ventricular arrhythmias in patients with severe CCC (Haedo et al., 1986). (6) Rosenbaum et al. (1987) found AMIO and flecainide to have comparable efficacy, each reducing ventricular arrhythmias in over 90% of patients (Rosenbaum et al., 1987). (7) Stein et al. (2018) conducted a meta-analysis of clinical trials, concluding that AMIO reduced VT episodes by 99.9%, ventricular premature beats by 93.1%, and ventricular couplets by 79%. These findings collectively support the efficacy of AMIO in reducing ventricular arrhythmia in CCC patients (Stein et al., 2018). In contrast, (8) Cardinalli-Neto et al. (2015) reported that AMIO therapy is independently associated with sustained VT inducibility during electrophysiologic study in patients with CCC. The authors note that this association may reflect AMIO as a marker of patients inherently at higher risk for malignant arrhythmias, rather than a causal effect (Cardinalli-Neto et al., 2015).

**TABLE 3:** Clinical studies evaluating amiodarone in patients with Chagas cardiomyopathy.

Author, Year, doi	IF*	Article type	N	Intervention	Follw-up	Aim	Endpoins: clinical effect of AMD	Side effects
Bellotti et al. (1983) doi: 10.1016/0002-9149(8390529-5).	American Journal of Cardiology (2,8)	Interventional, prospective, non-randomized	14	AMD, 900 to 1050 mg intravenous continuous infusion	1 day	Evaluate the effect of long-term AMD on ventricular tachycardia and ventricular premature beats;	Ventricular premature beats: 83.3% (10/12) of patients showed a reduction in the number of ventricular premature beats. Ventricular tachycardia: Ventricular tachycardia was suppressed in 33.3% (2/6) of patients.	28.6% (4/14)of patients had sinus bradycardia.
Rosenbaum et al. (1983) doi: 10.1016/0002-8703(8390022-4).	American Heart Journal (5,1)	Narrative clinical review	24	Patients received AMD (mean daily dose: 100 to 300 mg/day).	26 months	Evaluate the effect of long-term AMD treatment on CCC patients;	AMD totally and persistently suppressed extrasystolic couplets and ventricular tachycardia and reduced the number of persistent ventricular premature beats more than 93%	The incidence of: cornea microdeposits; hepatic dysfunction and Photosensitivity dermatitis were approximately 10 %
Chiale et al. (1984) doi: 10.1016/0002-8703(8490311-9).	International Journal of Cardiology (3.2)	Prospective, open-label, single-arm interventional study (non-randomized)	24	Patients received AMD (mean daily dose: 600 - 800 mg).	2 Years	Evaluate the effect of long-term AMD treatment on CCC patients;	AMD showed a remarkably high and sustained antiarrhythmic response 91% of the patients; AMD caused a decrease in propensy for recurrencies of ventriculat fibrilation (VF) and tachycardia (VT) and might prevent sudden death.	Corneal microdeposits without impairment of visual acuity; mild gastric discomfort; violaceus facial discoloration, thyroxicosis and bradycardia.
Carrasco et al. (1985) doi: 10.1016/0167-5273(8590238-4).	International Journal of Cardiology (3.2)	Randomized, double-blind, placebo-controlled crossover	9	Patients received AMD (mean daily dose: 200 mg).	2 weeks	Comparing efficacy of disopyramide versu AMD in patients with CCC;	Antiarrhythmic effect of amiodarone was total in 67%, partial in 11% and insignificant in 22% of patients with ventricular‘arrhythmias.	Not mentioned
Haedo et al. (1986) doi: 10.1016/s0735-1097(8680232-7).	Journal of the American College of Cardiology (21,7)	Prospective, placebo-controlled, within-subject crossover	14	Patients received AMD (mean daily dose: 800 mg).	1 week	Comparing the antiarrhythmic effect of verapamil, 17-monochlor-acetylajmaline, mexiletine and amiodarone in CCC patientes	AMD is clearly superior to other drugs for the treatment of potentially malignant ventricular arrhythmias in patients with severe chronic chagasic myocarditis.	Sisus bradycardia or transient sinoatrial block, gastrointestinal problems and corneal microdeposites.
Rosenbaum et al. (1987)	Archivos de Cardiologia de Mexico (0,6)	Randomized Controlled Trial with active comparator	81	Patients received AMD (mean daily dose: 400 to 800 mg/day).	60 days	Comparing the treatment with AMD and Flecainide in CCC patientes	The percentage reduction of premature ventricular contractions at days 9, 16 and 60 were 7.6%, 90.1% and 90.7% with AMD; AMD also reduce in 95.2% and 92.6% the in couplets and ventricular tachycardia, respectively.	AMD group, treatment was discontinued in three patients (one because of sustained ventricular tachycardia and two because of severe photosensitive dermatosis)
Cardinalli-Neto et al. (2006) doi: 10.1111/j.1540-8167.2007.00954.x.	Journal of Cardiovascular Electrophysiology (2,9)	Prospective observational cohort study	90	A single-chamber cardioverter defibrillator (ICD) was implanted in 60% of the patients, all of then were also treated with AMD (mean daily dose: 300 mg).	180 days	Determining predictors of all-cause mortality for Chagas’ disease in patients receiving ICD therapy.	Number of shocks per patient by day 30 is a powerful independent predictor of all-cause mortality for Chagas’ disease patients treated with ICD. Besides, the number of shocks per patient was much higher than what has been observed in non-Chagas’ disease patients, in spite of the fact that our patients received AMD.	Not mentioned
Ayub-Ferreira et al. (2013) doi:10.1371/journal.pntd.0002176.	Plos Neglected Tropical Diseases (3,4)	Subanalysis of prospectiveTrial: “REMADHE”	342 (Chagas: 55 versus 287 non-Chagas)	-	8 years	All-cause, heart failure and sudden death mortality;	AMD use, was independent risk factor for death from progressive heart failure.	None patient died from lung toxicity
Cardinalli-Neto et al. (2015) doi: 10.1016/j.ijcha.2015.10.001.	International Journal of Cardiology (3.2)	Retrospective observational cohort study	47	29% of the patients received AMD (mean daily dose: 300 mg).	6 years	Indentify independent predictors of inducible sustained ventricular tachycardia (VT) during electrophysiologic Study (EPS) in patients with CCC;	AMD therapy was an independent predictor of inducible sustained VT during EPS.	Not mentioned
Carmo et al. (2015) doi: 10.1016/j.ijcard.2015.04.061.	International Journal of Cardiology (3.2)	Retrospective nested case-control study within a prospective cohort	601 (AMD: 37 versus 564 non-AMD)	Patients received AMD (mean daily dose: 205 mg).	2 years	Indentify by PCR assays the impact of AMD treatment on the parasite load;	Using a well validated PCR assay to measure parasitic load in blood stream of ChD patients, was not demonstrate lower levels of *T. cruzi* parasitemia in habitual users of AMD.	Not mentioned
Carmo et al. (2018) doi: 10.1016/j.ijcard.2018.05.091.	International Journal of Cardiology (3.2)	Meta-analysis	598 (AMD: 115 versus 483 non-AMD)	-	-	Comparing efficacy of ICD versus medical treatment with AMD in patients with CCC;	Pooled data analysis did not show any difference in mortality outcomes between ICD and amiodarone treatment groups.	Not mentioned
Leite et al. (2003) doi: 10.1046/j.1540-8167.2003.02278.x.	Journal of Cardiovascular Electrophysiology (2,9)	Comparative Observational Study	115 (AMD: 78 versus sotalol: 37)	Patients received AMD (dose: 1000 mg/day for 7 to 10 days followed by 600 mg/day for 2 months and thereafter a maintence of 400 mg/day).	10 years	Evalueted the use of electrophysiologictestin to identify those who are at high risk despite Class III antiarrythmic drug therapy.	Total mortality didi not differ statistically among patientes treated with AMD (40,2%) and sotalol (36,0%).	7% of patients had to change the therapeutic strategy adopted at first due to the drug toxicity.
Gali et al. (2014) doi: 10.1093/europace/eut422.	EP Europace Journal (5,2)	Retrospective Cohort Study (Comparative Observational Study)	104 (ICD-plus-AMD: 76 versus AMD: 28)	Patients received AMD (mean daily dose: 300 - 400 mg).	5 Years	Comparing th eoutcomes of CCC patients with life-threatening ventricular arrhythmias (VAs), who were treated either with ICD implantation plus AMD or with AMD alone.	Compared with AMD-only therapy, ICD-plus-AMD reduced the risk of all-cause mortality and sudden death in CCC patients with life-threatening ventricular arrhytjmias.	Not mentioned
Rodríguez-Angulo et al. (2017) doi:10.1186/s12879-017-2324-x.	BMC Infectious Diseases (3,4)	Cross-Sectional Observational Study with Exploratory Biomarker Analysis	21 (AMD: 7 versus 14 untreated)	Patients received AMD (mean daily dose: 200 mg).	-	Determined the Th1/Th17 (IL-6, IL-2, TNF, IL-17 and IFN-γ) and Th2 (IL-4 and IL-10) serum profile in CCC patients according AMD treatment and arrhythmias.	Patients treated with AMD presented a significant decrease respect to the untreated ones in the relative levels of most of the cytokines analyzed (IL-17, IFN-γ, TNF, IL-4, IL-6 and IL-2)	Not mentioned
Fortes Silva et al. (2018) doi: 10.1111/pace.13384.	Pacing and Clinical Electrophysiology (1,4)	Cross-Sectional Observational Study	66 (untreated: 27 versus AMD: 16 versus health control: 23)	Patients received AMD (mean daily dose: 200 mg).	6 months	Evalueted the autonomic cardiac modulation in patients with CCC undergoing chronic amiodarone therapy;	Patients with CCC using AMD had changes in heart rate variability (HRV) suggestive of an offset in the sympatho-vagal balance with a vagal modulation predominance; the increase in the complexity of HRV strongly suggest that AMD may have a cardioprotective effect, which could increase the survival of these patients.	Not mentioned
Stein et al. (2018) doi: 10.1371/journal.pntd.0006742.	Plos Neglected Tropical Diseases (3,4)	Meta-analysis	-	Patients received AMD (mean daily dose: 200 - 1200 mg).	1 day to 27 months	Assess the effect of amiodarone in patients with Chagas cardiomyopathy.	AMD reduced the number of ventricular tachycardia episodes in 99.9%, ventricular premature beats in 93.1% and the incidence of ventricular couplets in 79%. AMD is effective in reducing ventricular arrhythmias, but there is no evidence for hard endpoints (sudden death, hospitalization).	Corneal microdeposits; gastrointestinal events; sinus bradycardia and dermatological events
Sousa et al. (2019) doi: 10.1590/0037-8682-0386-2019.	Revista da Sociedade Brasileira de Medicina Tropical (1,4)	Cross-Sectional Observational Study	40 (AMD: 11 versus 29 untreated)	Patients received AMD.	-	Evaluated the profile of Th1 and Th17 cytokines and IL-17, TNF-α, and IFN-γ expressions in different stages of CCC	Patients using AMD presented higher serum TNF-α concentrations	Not mentioned
Martinelli-Filho et al. (2024) doi: 10.1001/jamacardio.2024.3169.	JAMA Cardiology (24)	Open-label Randomized Controlled Trial	323 (AMD:166 versus 157 ICD)	Patients were randomized 1:1 to receive ICD or AMD (with a loading dose of 600 mg after randomization).	6 years	To test the hypothesis that ICD is more effective than AMD therapy for primary prevention of all-cause mortality in patients with CCC and moderate to high mortality risk, assessed by the Rassi score.	ICD did not reduce the risk of all-cause mortality. However, ICD significantly reduced the risk of SCD, pacing need, and heart failure hospitalization compared with AMD therapy.	Not mentioned

*Impact factors are based on data from 2024.

According to the 2023 SBC Guideline on Chagas Cardiomyopathy, AMIO is strongly recommended for treatment of symptomatic VEs in the absence of AV conduction abnormalities, ventricular dysfunction, segmental wall motion abnormalities, or myocardial fibrosis. However, for patients with sustained VT and left ventricular ejection fraction (LVEF) lower than 40%, the recommendation for AMIO use is classified as conditional, according to the GRADE system (Grading of Recommendations, Assessment, Development, and Evaluations) (Marin-Neto et al., 2023; Guyatt et al., 2011).

Evidence from preclinical and clinical studies demonstrate that AMIO effectively reduces ventricular arrhythmias in CCC, supporting its translational relevance; however, heterogeneity in study designs and patient characteristics, along with occasional reports of arrhythmia persistence or bradycardia, highlight the need for further controlled studies to fully establish efficacy and safety.

### Trypanocidal activity

This review includes nine articles that evaluated the trypanocidal effects of AMIO in preclinical models and in patients with CCC, with trypanocidal activity defined as the reduction of circulating parasites in peripheral blood and/or a decrease in overall parasitic load (Benaim et al., 2006; Carmo et al., 2018; Barbosa et al., 2022a; Barbosa et al., 2024; Benaim et al., 2012; Bellera et al., 2013; Veiga-Santos et al., 2012; Barbosa et al., 2022b; Adesse et al., 2011). The repositioning of AMIO as a potential trypanocidal agent was first proposed by (1) Benaim et al. (2006), who demonstrated its efficacy against intracellular parasites *in vitro* and in a mouse model of acute *T. cruzi* infection (Benaim et al., 2006). Later, (2) Benaim et al. (2012) compared AMIO with its derivative dronedarone and observed that dronedarone exhibited greater antiparasitic activity, suggesting that AMIO and its derivatives are privileged chemical scaffolds for anti-*T. cruzi* drug development (Benaim et al., 2012). These studies also elucidated AMIO’s mechanism of action, which includes disruption of Ca2+ homeostasis and inhibition of oxidosqualene cyclase, a key enzyme in parasite sterol biosynthesis (Benaim et al., 2006). (3) Bellera et al. (2013) further proposed that AMIO’s trypanocidal effect may also be partially attributed to its inhibition of cruzipain activity, a cysteine protease essential for parasite survival (Bellera et al., 2013). In agreement, our group in **(4)** Barbosa et al. (2022a) reproduced the reduction of peak parasitemia in mice treated with AMIO during acute *T. cruzi* infection, reinforcing its potential efficacy during the acute phase (Barbosa et al., 2022a).

(5) Veiga-Santos et al. (2012) described ultrastructural changes in intracellular amastigotes treated with AMIO, such as increased formation of autophagosomes and plasma membrane projections, Golgi disruption, and lipid body accumulation (Veiga-Santos et al., 2012). Similar observations regarding lipid body accumulation were made by our group in (6) Barbosa et al. (2022b). In contrast, (7) Adesse et al. (2011) reported mitochondrial swelling, reservosome formation, and kinetoplast disorganization. Variations in these findings likely reflect differences in experimental protocols, such as AMIO concentration, treatment duration, and infection phase (Table 2) (Adesse et al., 2011).

The efficacy of AMIO in chronic infection remains uncertain. In a murine model of chronic *T. cruzi* infection, our group in (8) Barbosa et al. (2024) found that AMIO treatment did not reduce parasitemia or tissue parasite load in the heart (Barbosa et al., 2024). Similarly, in a clinical study, (9) Carmo et al. (2015), using a PCR-based method to quantify parasitic DNA in the blood, did not detect parasitemia in CCC patients routinely treated with AMIO. However, the study had several limitations: it was retrospective in design, included a small number of AMIO users, and lacked detailed information on treatment duration. Therefore, the authors do not exclude the possibility of a trypanocidal effect of AMIO in patients with CCC and highlight the need for more rigorous studies (Carmo et al., 2015).

Overall, preclinical studies demonstrate that AMIO exerts trypanocidal effects, reducing parasitemia and/or parasitic load; however, these effects have not been consistently observed in chronic murine models or in patients with CCC, highlighting the need for further clinical research.

### Combined therapy

Eight studies investigated the efficacy of AMIO in combination with other pharmacological agents to improve treatment outcomes in CCC (Benaim et al., 2006; Madigan et al., 2019; Barbosa et al., 2022a; Barbosa et al., 2024; Barbosa et al., 2022b; Zao et al., 2019; Sass et al., 2019; Lourenço et al., 2018) (Table 2). The earliest study on AMIO combination therapy was conducted by (1) Benaim et al. (2006), who evaluated its synergy with posaconazole, a triazole antifungal agent. Both drugs are ergosterol biosynthesis inhibitors. The authors observed a synergistic antiparasitic effect, which they attributed to the combination of two complementary mechanisms: disruption of intracellular Ca2+ homeostasis and inhibition of ergosterol biosynthesis (Benaim et al., 2006).

Subsequent studies by (2) Madigan et al. (2019), (3) Zao et al. (2019), and (4) Sass et al. (2019) evaluated the combination of AMIO and itraconazole (Madigan et al., 2019; Zao et al., 2019; Sass et al., 2019). *In vitro*, the AMIO plus itraconazole combination was more effective against *T. cruzi* than either drug alone or Bz. Infected Vero cells and human cardiomyocytes (hiPSC-CMs) treated with the combination also showed preserved cell integrity (Sass et al., 2019). *In vivo*, naturally chronically infected dogs treated with AMIO plus itraconazole had improved cardiac functional parameters, including increased fractional shortening, reduced ventricular wall thickening, resolution of valvular regurgitation, and improved septal wall motion. Additionally, parasitemia measured by RT-PCR was reduced to low or undetectable levels, and survival rates improved (Madigan et al., 2019; Zao et al., 2019).

The interaction between AMIO and Bz, a nitro-derivative compound, was also investigated. (5) Lourenço et al. (2018) first tested the combination in *T. cruzi* epimastigotes and found no pharmacological interference - neither synergy nor antagonism. Under these experimental conditions, AMIO exhibited trypanocidal activity comparable to that of Bz (Lourenço et al., 2018). In an *in vitro* model, our group in (6) Barbosa et al. (2022b) confirmed this result in mammalian-infective forms (trypomastigotes and amastigotes). However, in *T. cruzi-*infected cardiac cells, the combination appeared to exert a protective effect, improving host cell cytoskeletal integrity (Barbosa et al., 2022b).

*In vivo* studies yielded more promising outcomes. In a mouse model of acute infection, our group in (7) Barbosa et al. (2022a) demonstrated that the AMIO plus Bz combination enhanced parasite clearance, increased survival, and improved expression of connexin-43 (Cx-43) in cardiac tissue. The combination also attenuated heart inflammation by lowering levels of monocyte chemoattractant protein-1 (MCP-1), interleukin 6 (IL-6), and tumor necrosis factor (TNF), and corrected cardiac electrical abnormalities (Barbosa et al., 2022a). Further, in a murine model of chronic infection, our group in (8) Barbosa et al. (2024) reported that AMIO plus Bz was the most effective intervention for mitigating ventricular dysfunction. It also modulated several key pathogenic pathways in CCC, including TNF production, reactive oxygen species production, fibronectin deposition, and Cx-43 expression in cardiac tissue (Barbosa et al., 2024). Finally, although the BENEFIT (Randomized Trial of Benznidazole for Chronic Chagas’ Cardiomyopathy) trial demonstrated that Bz monotherapy does not prevent progression of CCC in patients with established cardiomyopathy, *post hoc* analysis (Morillo et al., 2015; Rassi et al., 2017) suggested that concurrent use of AMIO and Bz could reduce cardiovascular hospitalization and mortality rates, indicating a potential beneficial role for the combination therapy in advanced stages of CCC.

Current evidence demonstrates that preclinical studies suggest AMIO, in combination with other pharmacological agents, may enhance anti-*T. cruzi* efficacy, improve cardiac function, and reduce pathogenic processes in CCC; however, no clinical data are currently available, highlighting the need for well-designed studies to confirm the translational potential of these combination therapies.

### Immunomodulatory effects

Our review includes four studies that assessed the immunomodulatory properties of AMIO in patients with CCC (Barbosa et al., 2022a; Barbosa et al., 2024; Rodríguez-Angulo et al., 2017; Sousa et al., 2019). In preclinical studies using murine models of both acute and chronic *T. cruzi* infection, AMIO was shown to reduce myocardial inflammation, as evidenced by quantification of inflammatory infiltrates in histopathological analysis (Barbosa et al., 2022a; Barbosa et al., 2024) (Table 2). (1) In the acute phase, our group in Barbosa et al. (2022a) reported that AMIO significantly decreased levels of MCP-1in heart tissue of *T. cruzi* infected-mice, compared to untreated infected mice (Barbosa et al., 2022a). In the chronic phase, in (2) Barbosa et al. (2024) we demonstrated that AMIO not only reduced cardiac inflammation but also led to a general decrease in peripheral leukocyte counts, particularly affecting lymphocyte populations (Barbosa et al., 2024).

In clinical studies involving CCC patients, (3) Rodríguez-Angulo et al. (2017) found that AMIO treatment significantly reduced the serum concentrations of several key cytokines, as measured by fluorescent bead-based flow cytometry. Specifically, they observed reductions in both pro-and anti-inflammatory cytokines, including interleukin 17 (IL-17), interferon gamma (IFN-γ), TNF, interleukin 4 (IL-4), IL-6, and interleukin 2 (IL-2) (Rodríguez-Angulo et al., 2017). Conversely, (4) Sousa et al. (2019) reported higher serum levels of TNF-α in CCC patients undergoing AMIO therapy, indicating variability in the immunological effects and highlighting the need for further studies to elucidate the pathways through which AMIO influences cytokine production in CCC (Table 3) (Sousa et al., 2019).

Collectively, both preclinical and clinical studies suggest that AMIO can modulate immune responses in CCC, reducing myocardial inflammation and affecting cytokine profiles; however, observed variability between studies underscores the need for further research to clarify the mechanisms and clinical significance of these immunomodulatory effects.

## Discussion

AMIO is traditionally classified as a class III antiarrhythmic, primarily due to its modulation of potassium channels, which leads to the prolongation of action potential duration, repolarization, and refractoriness, according to the Vaughan-Williams classification (Mujović et al., 2020). However, AMIO is distinctive in that it exhibits both pharmacological and clinical effects spanning all four antiarrhythmic classes outlined in the Vaughan-Williams classification. These include sodium channel blockade (class I), noncompetitive inhibition of alpha- and beta-adrenergic receptors (class II), and calcium channel blockade (class IV) (Nattel, 1993).

In cardiopathies of diverse etiologies, the comparison of AMIO versus placebo or control and versus other antiarrhythmic agents for primary prevention of cardiac death was evaluated in the ATMA meta-analysis (1997), which used individual patient data from eight randomized controlled trials (RCTs) conducted after acute myocardial infarction (EMIAT, CAMIAT, GEMICA, PAT, SSSD, BASIS, Hockings et al., 1987, and CAMIAT-P; total of 5,101 participants, 78%) and five studies including patients with congestive heart failure (CHF-STAT, GESICA, EPAMSA, Nicklas et al., 1991; and Hamer et al., 1989; total of 1,452 participants, 22%) (Effect of prophylactic amiodarone on, 1997). This meta-analysis showed a 13% reduction in the risk of total death (p = 0.03) and a 29% reduction in the risk of arrhythmogenic sudden death (p = 0.0003) with AMIO. It is important to note that only the GESICA study identified patients with CD within this cohort, representing 0.7% of the total population (6,553 participants). Moreover, only 21 individuals with CD received amiodarone treatment, corresponding to 0.3% of the overall ATMA trial cohort (Doval et al., 1994).

Furthermore, a meta-analysis conducted by Sim et al. (1997) incorporated data from the same 13 studies included in the ATMA trial, as well as two additional studies involving survivors of cardiac arrest or supraventricular tachycardia (CASCADE; total of 228 participants and ASSG; total of 59 participants). The analysis found that AMIO reduced all-cause mortality by approximately 19% (p < 0.01), with more significant reductions in cardiac mortality (23%, p < 0.001) and sudden death (30%, p < 0.001) (Sim et al., 1997). Therefore, in cardiopathies of various etiologies, the antiarrhythmic effects of AMIO and its role in the primary prevention of mortality are strongly supported by current literature (Marin-Neto et al., 2023).

In another meta-analysis, Claro et al. (2015) also analyzed treatment with AMIO for primary prevention (17 studies: Bardy et al., 2005; Biswas et al., 1996; BASIS; CAMIAT pilot; CAMIAT; Ceremuzynski et al., 1992; OPTIC; GESICA; GEMICA; Fournier et al., 1989; EPAMSA; CASCADE; Hamer et al., 1989; Harper et al., 1989; Hockings et al., 1987; EMIAT; Kovoor et al., 1999; ALPHEE; CASH; SSSD; Nicklas et al., 1991; STAT-CHF; Sousonis et al., 2014; Zehender et al., 1992; total of 8,383 participants). AMIO reduced sudden death (RR 0.76; 95% CI 0.66–0.88), cardiac mortality (RR 0.41; 95% CI 0.20–0.86), and all-cause mortality (RR 0.88; 95% CI 0.78–1.00) (Claro et al., 2015).

Few studies have included participants with CD in their cohorts. A meta-analysis published in 2018 aimed to evaluate the effect of AMIO in patients with CCC (Stein et al., 2018). This study included nine investigations (three before-and-after studies, five case series, and one randomized controlled trial). Two studies, involving a total of 38 patients, provided complete datasets, enabling an analysis of individual patient data (IPD). The main findings indicate that in 24-h Holter monitoring, AMIO led to a reduction in the number of ventricular tachycardia episodes by 99.9% (95% CI 99.8%–100%), ventricular premature beats by 93.1% (95% CI 82%–97.4%), and the incidence of ventricular couplets by 79% (RR 0.21, 95% CI 0.11–0.39). Therefore, AMIO was considered effective in reducing ventricular arrhythmias. However, in this study, there is no evidence supporting its impact on hard endpoints such as sudden death or hospitalization, and the quality of evidence analyzed ranged from moderate to very low (Stein et al., 2018).

Recently, Martinelli-Filho et al. (2024), in an open-label, randomized clinical trial named CHAGASICS, evaluated the hypothesis that ICD therapy is more effective than AMIO in preventing all-cause mortality in patients with CCC at moderate to high risk of mortality, as determined by the Rassi score. Patients were randomized 1:1, with 166 in the AMIO group and 157 in the ICD group. The rates of sudden cardiac death (SCD) (3.8% vs. 13.9%; HR, 0.25; P = 0.001), bradycardia requiring pacing (1.9% vs. 16.3%; HR, 0.10; P < 0.001), and heart failure hospitalization (8.9% vs. 16.9%; HR, 0.46; P = 0.01) were lower in the ICD group compared with the AMIO group. Thus, the authors concluded that ICD significantly reduced the risk of SCD, need for pacing, and heart failure hospitalization compared with AMIO therapy. It is important to note that this study has some limitations, such as the number of participants included. The study initially aimed to enroll 1,100 patients to provide 90% power to detect a significant difference between the ICD and AMIO groups. However, due to administrative issues, recruitment was halted at 323 participants, which caused a significant reduction in statistical power and the ability to identify meaningful differences between the groups (Martinelli-Filho et al., 2024).

In addition, in patients with an ICD, shocks are associated with increased morbidity, mortality, and decline in quality of life (Kheiri et al., 2019; Malik and Aronow, 2019). Therefore, AMIO may be used in combination with ICD and catheter ablation for the treatment of VT. The combination of ICD and AMIO significantly reduced VT recurrence and ICD shocks. Compared to AMIO, sotalol significantly increased VT recurrence and ICD shocks (Kheiri et al., 2019). AMIO remains the most effective antiarrhythmic drug for reducing appropriate and inappropriate shocks in patients with ICD (Malik and Aronow, 2019).

Despite the lack of robust evidence-based data regarding the efficacy of AMIO in patients with Chagas cardiomyopathy, this drug has been used for more than four decades. According to the SBC Guideline on the Diagnosis and Treatment of Patients with Cardiomyopathy of Chagas Disease, AMIO has been used in the clinical management of patients with CCC due to its high antiarrhythmic efficacy, low incidence of proarrhythmic and intolerable side effects - particularly when used at lower doses - and its good safety profile, even in patients with ventricular dysfunction. For these reasons, AMIO is currently considered the first-line drug for treating patients with CCC and high-risk ventricular arrhythmias (Marin-Neto et al., 2023).

Multiple pathophysiological mechanisms contribute to the progression of cardiac injury, with CCC pathogenesis involving both direct tissue damage induced by *T. cruzi* and indirect inflammatory and/or immune-mediated tissue injury (Marin-Neto et al., 2023). However, etiological treatment during the chronic phase with Bz presents significant limitations, as its efficacy varies according to patient age, immune status, parasite strain, and disease stage, with particularly low cure rates (8%–30%) reported in the late chronic phase (Marin-Neto et al., 2023; Bern, 2011). In this context, drug repurposing of compounds already approved for human use, such as AMIO, represents a promising therapeutic strategy for CCC, given that their safety and pharmacokinetic profiles have already been validated (Freire et al., 2025). Moreover, accumulating evidence suggests that this drug may contribute to parasite clearance, particularly when combined with Bz, and may also modulate the host immune response, potentially reducing tissue damage (Benaim et al., 2006; Barbosa et al., 2022a; Barbosa et al., 2024; Barbosa et al., 2022b).

In this review, we highlight the lack of robust clinical studies on the effect of AMIO in patients with CCC, as documented by Stein et al. (2018), whose meta-analysis using the GRADE framework reported the overall quality of evidence as ranging from moderate to very low, reflecting a clear gap in the current literature (Stein et al., 2018). Additionally, we identify relevant topics that may shed light on the role of AMIO in the treatment of CD, such as its potential trypanocidal and immunomodulatory effects, as well as more effective therapeutic alternatives, such as combined therapy with other drugs.

## References

[B1] Adesse D. Azzam E. M. Meirelles M. deN. Urbina J. A. Garzoni L. R. (2011). Amiodarone inhibits Trypanosoma cruzi infection and promotes cardiac cell recovery with gap junction and cytoskeleton reassembly *in vitro* . Antimicrob. Agents Chemotherapy 55 (1), 203–210. 10.1128/AAC.01129-10 21078932 PMC3019665

[B61] Amiodarone vs Sotalol Study Group (1989). Multicentre randomized trial of sotalol vs amiodarone for chronic malignant ventricular tachyarrhythmias. Eur. Heart J. 10 (8), 685–694. 10.1093/oxfordjournals.eurheartj.a059531 2676535

[B2] Aria M. Cuccurullo C. (2017). Bibliometrix: an R-tool for comprehensive science mapping analysis. J. Inf. 11 (4), 959–975. 10.1016/j.joi.2017.08.007

[B3] Ayub-Ferreira S. M. Mangini S. Issa V. S. Cruz F. D. Bacal F. Guimarães G. V. (2013). Mode of death on chagas heart disease: comparison with other etiologies. A subanalysis of the REMADHE prospective trial. PLoS Neglected Tropical Diseases 7 (4), e2176. 10.1371/journal.pntd.0002176 23638197 PMC3636047

[B4] Barbosa J. M. C. Pedra Rezende Y. de Melo T. G. de Oliveira G. Cascabulho C. M. Pereira E. N. G. D. S. (2022a). Experimental combination therapy with amiodarone and low-dose benznidazole in a mouse model of Trypanosoma cruzi acute infection. Microbiol. Spectrum 10 (1), e0185221. 10.1128/spectrum.01852-21 35138142 PMC8826820

[B5] Barbosa J. M. C. Pedra-Rezende Y. Pereira L. D. de Melo T. G. Barbosa H. S. Lannes-Vieira J. (2022b). Benznidazole and amiodarone combined treatment attenuates cytoskeletal damage in *trypanosoma cruzi*-infected cardiac cells. Front. Cellular Infection Microbiology 12, 975931. 10.3389/fcimb.2022.975931 36093188 PMC9452897

[B6] Barbosa J. M. C. Pedra-Rezende Y. Mata-Santos H. A. Vilar-Pereira G. Melo T. G. Ramos I. P. (2024). Preclinical evaluation of combined therapy with amiodarone and low-dose benznidazole in a mouse model of chronic Trypanosoma cruzi infection. Biomed. and Pharmacotherapy = Biomedecine and Pharmacotherapie 175, 116742. 10.1016/j.biopha.2024.116742 38754265

[B62] Bardy G. H. Lee K. L. Mark D. B. Poole J. E. Packer D. L. Boineau R. (2005). Amiodarone or an implantable cardioverter-defibrillator for congestive heart failure. N. Engl. J. Med. 352 (3), 225–237. 10.1056/NEJMoa043399 15659722

[B7] Bellera C. L. Balcazar D. E. Alberca L. Labriola C. A. Talevi A. Carrillo C. (2013). Application of computer-aided drug repurposing in the search of new cruzipain inhibitors: discovery of amiodarone and bromocriptine inhibitory effects. J. Chemical Information Modeling 53 (9), 2402–2408. 10.1021/ci400284v 23906322

[B8] Bellotti G. Silva L. A. Esteves Filho A. Rati M. de Moraes A. V. Ramires J. A. (1983). Hemodynamic effects of intravenous administration of amiodarone in congestive heart failure from chronic chagas' disease. Am. Journal Cardiology 52 (8), 1046–1049. 10.1016/0002-9149(83)90529-5 6637821

[B9] Benaim G. Sanders J. M. Garcia-Marchán Y. Colina C. Lira R. Caldera A. R. (2006). Amiodarone has intrinsic Anti-Trypanosoma cruzi activity and acts synergistically with posaconazole. J. Medicinal Chemistry 49 (3), 892–899. 10.1021/jm050691f 16451055

[B10] Benaim G. Hernandez-Rodriguez V. Mujica-Gonzalez S. Plaza-Rojas L. Silva M. L. Parra-Gimenez N. (2012). *In vitro* Anti-Trypanosoma cruzi activity of dronedarone, a novel amiodarone derivative with an improved safety profile. Antimicrob. Agents Chemotherapy 56 (7), 3720–3725. 10.1128/AAC.00207-12 22508311 PMC3393446

[B11] Bern C. (2011). Antitrypanosomal therapy for chronic chagas' disease. N. Engl. Journal Medicine 364 (26), 2527–2534. 10.1056/NEJMct1014204 21714649

[B12] Bern C. (2015). Chagas' disease. N. Engl. Journal Medicine 373 (19), 1882. 10.1056/NEJMc1510996 26535522

[B70] Biswas A. Dey S. K. Banerjee A. K. Roy S. Biswas P. K. Chowdhury G. K. (1996). Low-dose amiodarone in severe chronic heart failure. Indian Heart J. 48 (4), 361–364. 8908821

[B13] Cardinalli-Neto A. Greco O. T. Bestetti R. B. (2006). Automatic implantable cardioverter-defibrillators in chagas' heart disease patients with malignant ventricular arrhythmias. Pacing Clinical Electrophysiology PACE 29 (5), 467–470. 10.1111/j.1540-8159.2006.00377.x 16689840

[B14] Cardinalli-Neto A. Lorga-Filho A. M. Silva E. F. Lima R. P. Palmegiani E. Bestetti R. B. (2015). Clinical predictors of inducible sustained ventricular tachycardia during electrophysiologic study in patients with chronic chagas' heart disease. Int. Journal Cardiology. Heart and Vasculature 9, 85–88. 10.1016/j.ijcha.2015.10.001 28785714 PMC5497327

[B15] Carmo A. A. Rocha M. O. Silva J. L. Ianni B. M. Fernandes F. Sabino E. C. (2015). Amiodarone and Trypanosoma cruzi parasitemia in patients with chagas disease. Int. Journal Cardiology 189, 182–184. 10.1016/j.ijcard.2015.04.061 25897900 PMC4446230

[B16] Carmo A. A. L. de Sousa M. R. Agudelo J. F. Boersma E. Rocha M. O. C. Ribeiro A. L. P. (2018). Implantable cardioverter-defibrillator in chagas heart disease: a systematic review and meta-analysis of observational studies. Int. Journal Cardiology 267, 88–93. 10.1016/j.ijcard.2018.05.091 29871807

[B17] Carrasco H. A. Vicuña A. V. Molina C. Landaeta A. Reynosa J. Vicuña N. (1985). Effect of low oral doses of disopyramide and amiodarone on ventricular and atrial arrhythmias of chagasic patients with advanced myocardial damage. Int. Journal Cardiology 9 (4), 425–438. 10.1016/0167-5273(85)90238-4 3908329

[B63] Ceremuzynski L. Kleczar E. Krzeminska-Pakula M. Kuch J. Nartowicz E. Smielak-Korombel J. (1992). Effect of amiodarone on mortality after myocardial infarction: a double-blind, placebo-controlled, pilot study. J. Am. Coll. Cardiol. 20 (5), 1056–1062. 10.1016/0735-1097(92)90357-s 1401602

[B18] Charlier R. Delaunois G. Bauthier J. Deltour G. (1969). Recherches dans la série des benzofuranes. XL. Propriétés antiarythmiques de l'amiodarone. Cardiologia 54 (2), 83–90. 4190414

[B19] Chatelain E. (2016). Chagas disease research and development: is there light at the end of the tunnel? Comput. Structural Biotechnology Journal 15, 98–103. 10.1016/j.csbj.2016.12.002 28066534 PMC5196238

[B20] Chiale P. A. Halpern M. S. Nau G. J. Tambussi A. M. Przybylski J. Lázzari J. O. (1984). Efficacy of amiodarone during long-term treatment of malignant ventricular arrhythmias in patients with chronic chagasic myocarditis. Am. Heart Journal 107 (4), 656–665. 10.1016/0002-8703(84)90311-9 6702559

[B21] Claro J. C. Candia R. Rada G. Baraona F. Larrondo F. Letelier L. M. (2015). Amiodarone *versus* other pharmacological interventions for prevention of sudden cardiac death. Cochrane Database Systematic Reviews 2015 (12), CD008093. 10.1002/14651858.CD008093.pub2 26646017 PMC8407095

[B22] Cumpston M. Li T. Page M. J. Chandler J. Welch V. A. Higgins J. P. (2019). Updated guidance for trusted systematic reviews: a new edition of the cochrane handbook for systematic reviews of interventions. Cochrane Database Systematic Reviews 10 (10), ED000142. 10.1002/14651858.ED000142 31643080 PMC10284251

[B23] Doval H. C. Nul D. R. Grancelli H. O. Perrone S. V. Bortman G. R. Curiel R. (1994). Randomised trial of low-dose amiodarone in severe congestive heart failure. Grupo de Estudio de la Sobrevida en la Insuficiencia Cardiaca en Argentina (GESICA). Lancet London, Engl. 344 (8921), 493–498. 10.1016/s0140-6736(94)91895-3 7914611

[B24] Effect of prophylactic amiodarone on mortality after acute myocardial infarction and in congestive heart failure: meta-analysis of individual data from 6500 patients in randomised trials. Amiodarone trials meta-analysis investigators. (1997). Lancet London, Engl. 350(9089), 1417–1424. 9371164

[B25] Fortes Silva H. E. de Almeida R. S. Silveira D. B. Llaguno M. Resende L. A. P. R. Dias da Silva V. J. (2018). Cardiac autonomic modulation and long-term use of amiodarone in patients with chronic chagasic cardiopathy. Pacing Clinical Electrophysiology PACE 41 (7), 788–798. 10.1111/pace.13384 29781516

[B71] Fournier C. Brunet M. Bah M. Kindermans M. Boujon B. Tournadre P. (1989). Comparison of the efficacy of propranolol and amiodarone in suppressing ventricular arrhythmias following myocardial infarction. Eur. Heart J. 10 (12), 1090–1100. 10.1093/oxfordjournals.eurheartj.a059431 2691252

[B26] Freire E. S. da Silva L. P. Silva A. do C. Vaz de Castro P. A. S. de Araújo G. R. Otta D. A. (2025). New drugs and promising drug combinations in the treatment of chagas disease in Brazil: a systematic review and meta-analysis. Arch. Med. Res. 56, 103084. 10.1016/j.arcmed.2024.103084 39332069

[B27] Gali W. L. Sarabanda A. V. Baggio J. M. Ferreira L. G. Gomes G. G. Marin-Neto J. A. (2014). Implantable cardioverter-defibrillators for treatment of sustained ventricular arrhythmias in patients with Chagas' heart disease: comparison with a control group treated with amiodarone alone. Europace 16 (5), 674–680. 10.1093/europace/eut422 24481778

[B28] Greco O. T. Lorga A. M. Garzon S. A. Younan I. Bellini A. J. Bilaqui A. (1980). A amiodarona nas arritmias ventriculares da cardiopatia chagásica crónica [Amiodarone in ventricular arrhythmias of chronic Chagas cardiopathy]. Arq. Bras. Cardiol. 35 (2), 177–181. 7213094

[B29] Grupi C. J. Moffa P. J. Barbosa S. A. Sanches P. C. Barragan Filho E. G. Bellotti G. M. (1995). Holter monitoring in Chagas' heart disease. Sao Paulo Med. J. = Revista paulista de Med. 113 (2), 835–840. 10.1590/s1516-31801995000200015 8650484

[B30] Guyatt G. H. Oxman A. D. Kunz R. Brozek J. Alonso-Coello P. Rind D. (2011). GRADE guidelines 6. Rating the quality of evidence--imprecision. J. Clinical Epidemiology 64 (12), 1283–1293. 10.1016/j.jclinepi.2011.01.012 21839614

[B31] Haedo A. H. Chiale P. A. Bandieri J. D. Lázzari J. O. Elizari M. V. Rosenbaum M. B. (1986). Comparative antiarrhythmic efficacy of verapamil, 17-monochloracetylajmaline, mexiletine and amiodarone in patients with severe chagasic myocarditis: relation with the underlying arrhythmogenic mechanisms. J. Am. Coll. Cardiol. 7 (5), 1114–1120. 10.1016/s0735-1097(86)80232-7 3958370

[B64] Hamer A. W. Arkles L. B. Johns J. A. (1989). Beneficial effects of low dose amiodarone in patients with congestive cardiac failure: a placebo-controlled trial. J. Am. Coll. Cardiol. 14 (7), 1768–1774. 10.1016/0735-1097(89)90030-2 2685081

[B65] Hockings B. E. George T. Mahrous F. Taylor R. R. Hajar H. A. (1987). Effectiveness of amiodarone on ventricular arrhythmias during and after acute myocardial infarction. Am. J. Cardio. 60 (13), 967–970. 10.1016/0002-9149(87)90334-1 3673913

[B32] Kheiri B. Barbarawi M. Zayed Y. Hicks M. Osman M. Rashdan L. (2019). Antiarrhythmic drugs or catheter ablation in the management of ventricular tachyarrhythmias in patients with implantable Cardioverter-Defibrillators: a systematic review and meta-analysis of randomized controlled trials. Circulation. Arrhythmia Electrophysiology 12 (11), e007600. 10.1161/CIRCEP.119.007600 31698933

[B66] Kovoor P. Eipper V. Byth K. Cooper M. J. Uther J. B. Ross D. L. (1999). Comparison of sotalol with amiodarone for long-term treatment of spontaneous sustained ventricular tachyarrhythmia based on coronary artery disease. Eur. Heart Journal 20 (5), 364–374. 10.1053/euhj.1998.1279 10206383

[B33] Kumar S. Kumar S. Dhoble S. (2019). Quantitative and qualitative study of the research collaboration among scientists at the Indian Institute of Oilseeds Research. J. Inf. Syst. Manag. 9 (1), 20–28. 10.6025/jism/2019/9/1/20-28

[B34] Leite L. R. Fenelon G. Simoes A. Jr Silva G. G. Friedman P. A. de Paola A. A. (2003). Clinical usefulness of electrophysiologic testing in patients with ventricular tachycardia and chronic chagasic cardiomyopathy treated with amiodarone or sotalol. J. Cardiovascular Electrophysiology 14 (6), 567–573. 10.1046/j.1540-8167.2003.02278.x 12875414

[B35] Lidani K. C. F. Andrade F. A. Bavia L. Damasceno F. S. Beltrame M. H. Messias-Reason I. J. (2019). Chagas disease: from discovery to a worldwide health problem. Front. Public Health 7, 166. 10.3389/fpubh.2019.00166 31312626 PMC6614205

[B36] Lourenço A. M. Faccini C. C. Costa C. A. J. Mendes G. B. Fragata Filho A. A. (2018). Evaluation of *in vitro* Anti-Trypanosoma cruzi activity of medications benznidazole, amiodarone hydrochloride, and their combination. Rev. Soc. Bras. Med. Trop. 51 (1), 52–56. 10.1590/0037-8682-0285-2017 29513842

[B37] Madigan R. Majoy S. Ritter K. Luis Concepción J. Márquez M. E. Silva S. C. (2019). Investigation of a combination of amiodarone and itraconazole for treatment of American trypanosomiasis (Chagas disease) in dogs. J. Am. Veterinary Med. Assoc. 255 (3), 317–329. 10.2460/javma.255.3.317 31298647

[B38] Malik A. H. Aronow W. S. (2019). Prevention of recurrent ventricular tachycardia in patients with implantable cardioverter Defibrillators-A network meta-analysis. Am. Journal Therapeutics 26 (4), e469–e480. 10.1097/MJT.0000000000000928 30946044

[B39] Marin-Neto J. A. Rassi A. Jr Oliveira G. M. M. Correia L. C. L. Ramos Júnior A. N. Luquetti A. O. (2023). SBC Guideline on the Diagnosis and Treatment of Patients with Cardiomyopathy of Chagas Disease - 2023. Diretriz da SBC sobre Diagnóstico e Tratamento de Pacientes com Cardiomiopatia da Doença de Chagas – 2023. Arq. Bras. Cardiol. 120 (6), e20230269. 10.36660/abc.20230269 37377258 PMC10344417

[B40] Martinelli Filho M. De Siqueira S. F. Moreira H. Fagundes A. Pedrosa A. Nishioka S. D. (2000). Probability of occurrence of life-threatening ventricular arrhythmias in Chagas' disease *versus* non-Chagas' disease. Pacing Clinical Electrophysiology PACE 23 (11 Pt 2), 1944–1946. 10.1111/j.1540-8159.2000.tb07058.x 11139963

[B41] Martinelli-Filho M. Marin-Neto J. A. Scanavacca M. I. de Paola A. A. V. Medeiros P. T. J. Owen R. (2024). Amiodarone or implantable cardioverter-defibrillator in Chagas cardiomyopathy: the CHAGASICS randomized clinical trial. JAMA Cardiology 9 (12), 1073–1081. 10.1001/jamacardio.2024.3169 39356542 PMC11447631

[B42] Morillo C. A. Marin-Neto J. A. Avezum A. Sosa-Estani S. Rassi A. Jr Rosas F. (2015). Randomized trial of benznidazole for chronic Chagas' cardiomyopathy. N. Engl. Journal Medicine 373 (14), 1295–1306. 10.1056/NEJMoa1507574 26323937

[B43] Mujović N. Dobrev D. Marinković M. Russo V. Potpara T. S. (2020). The role of amiodarone in contemporary management of complex cardiac arrhythmias. Pharmacol. Research 151, 104521. 10.1016/j.phrs.2019.104521 31756386

[B44] Nattel S. (1993). Comparative mechanisms of action of antiarrhythmic drugs. Am. Journal Cardiology 72 (16), 13F–17F. 10.1016/0002-9149(93)90959-g 8237825

[B67] Nicklas J. M. McKenna W. J. Stewart R. A. Mickelson J. K. Das S. K. Schork M. A. (1991). Prospective, double-blind, placebo-controlled trial of low-dose amiodarone in patients with severe heart failure and asymptomatic frequent ventricular ectopy. Am. Heart Journal 122 (4 Pt 1), 1016–1021. 10.1016/0002-8703(91)90466-u 1927852

[B45] Nunes M. C. P. Beaton A. Acquatella H. Bern C. Bolger A. F. Echeverría L. E. (2018). Chagas cardiomyopathy: an update of Current clinical knowledge and management: a scientific statement from the American heart Association. Circulation 138 (12), e169–e209. 10.1161/CIR.0000000000000599 30354432

[B46] Page M. J. McKenzie J. E. Bossuyt P. M. Boutron I. Hoffmann T. C. Mulrow C. D. (2021). The PRISMA 2020 statement: an updated guideline for reporting systematic reviews. BMJ Clin. Research ed. 372, n71. 10.1136/bmj.n71 33782057 PMC8005924

[B47] Rassi A. Jr Marin J. A. Rassi A. (2017). Chronic chagas cardiomyopathy: a review of the main pathogenic mechanisms and the efficacy of aetiological treatment following the BENznidazole evaluation for interrupting trypanosomiasis (BENEFIT) trial. Memorias Do Inst. Oswaldo Cruz 112 (3), 224–235. 10.1590/0074-02760160334 28225900 PMC5319366

[B48] Ribeiro A. L. P. Marcolino M. S. Prineas R. J. Lima-Costa M. F. (2014). Electrocardiographic abnormalities in elderly chagas disease patients: 10-Year Follow-Up of the bambui cohort Study of aging. J. Am. Heart Assoc. 3 (1), e000632. 10.1161/JAHA.113.000632 24510116 PMC3959704

[B49] Rodríguez-Angulo H. Marques J. Mendoza I. Villegas M. Mijares A. Gironès N. (2017). Differential cytokine profiling in Chagasic patients according to their arrhythmogenic-status. BMC Infectious Diseases 17 (1), 221. 10.1186/s12879-017-2324-x 28327099 PMC5361844

[B50] Rosenbaum M. B. Chiale P. A. Haedo A. Lázzari J. O. Elizari M. V. (1983). Ten years of experience with amiodarone. Am. Heart Journal 106 (4 Pt 2), 957–964. 10.1016/0002-8703(83)90022-4 6613843

[B51] Rosenbaum M. Posse R. Sgammini H. Núñez Burgos J. Chiale P. A. Pastori J. D. (1987). Comparative multicenter clinical study of flecainide and amiodarone in the treatment of ventricular arrhythmias associated with chronic Chagas cardiopathy. Arch. del Inst. Cardiol. Mex. 57 (4), 325–330. 2445316

[B52] Sass G. Madigan R. T. Joubert L. M. Bozzi A. Sayed N. Wu J. C. (2019). A combination of itraconazole and amiodarone is highly effective against *Trypanosoma cruzi* infection of human stem cell-derived cardiomyocytes. Am. Journal Tropical Medicine Hygiene 101 (2), 383–391. 10.4269/ajtmh.19-0023 31219005 PMC6685576

[B53] Sim I. McDonald K. M. Lavori P. W. Norbutas C. M. Hlatky M. A. (1997). Quantitative overview of randomized trials of amiodarone to prevent sudden cardiac death. Circulation 96 (9), 2823–2829. 10.1161/01.cir.96.9.2823 9386144

[B54] Sousa R. C. Deus D. B. Costa T. Á. D. Silva M. V. D. Rodrigues Junior V. Correia D. (2019). Correlation between the cytokine profile and anticongestive medication in patients with chronic chagasic cardiopathy. Rev. Soc. Bras. Med. Trop. 52, e20190386. 10.1590/0037-8682-0386-2019 31800924

[B68] Sousonis V. Kaldara E. Pantsios C. Repasos E. Kapelios C. Nana E. (2014). PVC suppression with amiodarone is associated with improvement in systolic function: a prospective, randomized study. Eur. J. Heart Fail. 16 (Suppl. 2), 28.

[B55] Stein C. Migliavaca C. B. Colpani V. da Rosa P. R. Sganzerla D. Giordani N. E. (2018). Amiodarone for arrhythmia in patients with chagas disease: a systematic review and individual patient data meta-analysis. PLoS Neglected Tropical Diseases 12 (8), e0006742. 10.1371/journal.pntd.0006742 30125291 PMC6130878

[B56] Tavolinejad H. Soltani D. Zargaran A. Rezaeizadeh H. Vasheghani-Farahani A. (2019). The story of amiodarone. Eur. Heart Journal 40 (33), 2758–2759. 10.1093/eurheartj/ehz583 31505605

[B57] Van Eck N. J. Waltman L. (2010). Software survey: vosviewer, a computer program for bibliometric mapping. Scientometrics 84 (2), 523–538. 10.1007/s11192-009-0146-3 20585380 PMC2883932

[B58] Veiga-Santos P. Barrias E. S. Santos J. F. de Barros Moreira T. L. de Carvalho T. M. Urbina J. A. (2012). Effects of amiodarone and posaconazole on the growth and ultrastructure of Trypanosoma cruzi. Int. Journal Antimicrobial Agents 40 (1), 61–71. 10.1016/j.ijantimicag.2012.03.009 22591838

[B59] World Health Organization (2025). Chagas disease (American trypanosomiasis). Geneva: World Health Organization. Available online at: https://www.who.int/news-room/fact-sheets/detail/chagas-disease-(american-trypanosomiasis) (Accessed 12 October 2025).

[B60] Zao C. L. Yang Y. C. Tomanek L. Cooke A. Berger R. Chien L. C. (2019). PCR monitoring of parasitemia during drug treatment for canine chagas disease. J. Veterinary Diagnostic Investigation Official Publication Am. Assoc. Veterinary Laboratory Diagnosticians, Inc 31 (5), 742–746. 10.1177/1040638719868508 31378166 PMC6727115

[B69] Zehender M. Faber T. Furtwängler A. Hohnloser S. Meinertz T. Just H. (1992). Risikostratifikation und Langzeittherapie mit Amiodaron bei Patienten mit idiopathischer dilativer Kardiomyopathie [Risk stratification and long-term therapy with amiodarone in patients with idiopathic dilated cardiomyopathy]. Z. fur Kardiologie 81 (12), 704–709. 1492440

